# News from the Societies

**Published:** 1962-01

**Authors:** 


*PLYMOUTH*

### *News from Societies*

B.M.A. (Barnstaple Division)

*Hon Secretary:* Dr. D. T. C. Barlow, 110 Boutport Street, Barnstaple

19th Jan.: **Lecture on "The Use of Steroid Therapy and Other Recent Advances in Treatment".**

10th Feb.: **Talk on "Recent Advances in the Chemotherapy of Cancer".**

3rd Mar.: **Talk on "Patterns of Injury in Homicide".**

28th Mar.: **Clinical Meeting.**

26th Apr.: **Discussion** of the **Annual Report,** followed by films.

30th May: **Annual General Meeting.**

PLYMOUTH MEDICAL SOCIETY

*Hon. Secretary:* Dr. Bernard Peck, North Friary House, Greenbank Terrace, Plymouth.

19th Jan., at 8.30 p.m.: **"Small Boat across the Ocean"** by Dr. D. H. Lewis, at North Friary House.

9th Feb., at 8.30 p.m.: Short papers by Dr. J. N. Montgomery, Dr. D. Razzak, Dr. W. Homer Smith, at North Friary House.

23rd Feb., at 8.30 p.m.: **Clinical Evening** (cases from 8 p.m.) at Royal Naval Hospital.

8th Mar., at 8.30 p.m. **"The Mental Health Act 1959".** Joint discussion meeting with the Incorporated Law Society of Plymouth, at North Friary House.

23rd Mar., at 8.30 p.m.: **Clinical Evening.** Symposium on **"Hiatus hernia"** (cases from 8 p.m.), at Freedom Fields Hospital.

2nd May, at 3. p.m.: Visit to the Ambrosia Ltd. Creamery. (Members' ladies also invited), at Lifton, Devon.

7th May, at 8.30 p.m.: **Annual General Meeting,** at North Friary House.

SOUTH SOMERSET MEDICAL SOCIETY

*Hon. Secretary:* Dr. S. T. Pickles, 68 Hendford, Yeovil.

18th Jan.: **"Miscellaneous Hazards to the Newborn Infant",** by Dr. Vulliamy.

15th Mar.: **"The Pulmonary Circulation and Heart Disease",** by Dr. Wood.

### TORQUAY AND DISTRICT MEDICAL SOCIETY

*Hon. Secretaries:* Dr. D. K. McTaggart, Health Department, St. Marychurch Town Hall, Torquay, and Dr. J. B. Taylor, Corner Place, Dartmouth Road, Paignton.

18th Jan., at 8.30 p.m.: **"Rheumatoid Arthritis",** by Dr. G. D. Kersley.
4th Feb., Sunday, at 11 a.m.: **Clinical Meeting.**
15th Feb., at 8.30 p.m. **"Family Practice in the U.S.S.R. To-day",** by Dr. John Hunt.
4th Mar., Sunday, at 11 a.m. **Clinical Meeting.**
15th Mar., at 8.30 p.m.: **"The Significance of Haematuria",** by Mr. Hugh Gerrier.
1st Apr., Sunday, at 11 a.m.: **Clinical Meeting.**
12th Apr., at 8 p.m.: **Clinical Evening.**
17th May, at 8.30 p.m.: **Annual General Meeting.**

The Sunday Clinical Meetings will be held at the Torbay Hospital and will last about an hour. Please meet in the Board Room where coffee will be served from 10.45 a.m. Will any member who wishes to show a case please inform Mr. Monks or Dr. D. MacTaggart not later than the Thursday prior to the meeting.

